# Notes and News

**Published:** 1901-09-14

**Authors:** 


### Notes and News.

The Oxford Eye Hospital has received a munificent gift of £3,000 from an anonymous donor.

The Dental Hospital of London has received the sum of £500 from the executors of the late Richard Bowerman West, Esq.

Professor Niels Finsen has, in conjunction with Professor Pawlow, of St. Petersburg, been awarded the Nobel Prizes of about £11,000.

The Metropolitan Hospital Saturday is fixed for October 12th this year. Judging from the amount collected up to the present time, the present year will be a very good one for the Fund.

The steamship *Rameses*, of the Moss line, bound for Liverpool, has been refused entrance at Malta and Gibraltar, owing to the existence of plague at Alexandria, and one fatal case has occurred on board.

The lot of the evil-doer is gradually and continuously being improved. During the present month a better dietary is to come into force in our gaols. In many cases meat and cocoa are to take the place of porridge.

It is expected that the central block of the new Sheffield Royal Infirmary will be completed by Christmas. £1,900 is still required to complete the £10,000 which, when secured, will entitle the hospital to receive £2,000 promised by the Town Trustees.

The Local Government Board have issued a circular to the various local authorities announcing the appointment of the Tuberculosis Commission, but adding that they are "desirous of impressing upon the local authorities concerned that, pending the investigations and report of the Royal Commission, there should be no relaxation on their part or on that of their officers in the taking of proper measures for dealing with milk from tuberculous cows and with tuberculous meat, which may be intended as food for man." This is as it should be.

A legacy of £40,000 has been left by Monsieur Brémond for the purpose of supporting an institution which provides for the early rearing of children born in the maternity departments of the hospitals at Lyons.

The fifth International Congress of Physiology opens on the 17th inst. at Turin. Professor Sherrington, of Liverpool, is a British representative on the committee, and the local secretary is Dr. Z. Treves, 30 Corso Raffaelo, Turin.

We referred recently to the action of the Halifax Board of Guardians in not re-electing Dr. Dolan as Medical Officer at the Poor Law Infirmary. The medical men of Halifax have shown their sympathy with Dr. Dolan by condoling with him on the want of recognition shown him after 30 years' service.

The recently published vital statistics of Sydney show the death-rate of the city to be a remarkably low one, only reaching as it does 12·39 per thousand population. This low death-rate has been maintained in spite of a visitation of the plague and an unusual prevalence of typhoid fever. During the past decade tuberculosis shows a marked diminution in Sydney.

The admirable work of assisting officers and nurses invalided home from South Africa which Lady Dudley has carried out for so long quietly and thoroughly is known to apparently few. She is now appealing to the public for assistance, which she has not found necessary to do before. Now that her work must become more widely known it is to be hoped that a warm appreciation of her efforts will be shown by a generous response to her appeal.

The report of the Committee on Navy Rations is an interesting document. Perhaps the recommendation as to a change in the hours of meals is the most important. At present the hours are 5 a.m., noon, and 4.15 p.m. The most strenuous advocate of a reasonable lapse of time between meals must admit that these regulations are severe in the extreme. The new arrangements proposed embrace: Chocolate at 5 a.m., breakfast at 8 a.m., tea at 4.15, and supper at 7.30, with a corresponding increase of leisure time for the consumption of the additional diet.

We much regret that in consequence of age and pulmonary disease Mr. William Tallack, the secretary of the Howard Association for 35 years, has found it necessary to resign that office. There may be some who imagine that the great work initiated by John Howard is a matter of merely historical interest. This is not so. Wherever human beings are placed at the mercy of their fellows, tyranny and abuses will creep in, and it requires the constant vigilance of men like Mr. Tallack, and of bodies like the Howard Association, to keep the public conscience awake on the subject. A quite recent paper by Mr. Tallack drew attention in forcible terms to the horrors which are still being perpetrated in the convict camps in the southern portion of the United States, and, coincident with the announcement of his retirement, comes an able letter from him on the subject of "British Liberty and Conventional Inspection." There is still plenty to be done for the protection of those who from any cause are under lock and key.

